# Near neighbour analysis of variant cell lines derived from the promyeloid cell line HL60.

**DOI:** 10.1038/bjc.1988.128

**Published:** 1988-06

**Authors:** C. M. Bunce, J. M. Lord, A. K. Wong, G. Brown

**Affiliations:** Department of Immunology, University of Birmingham, Edgbaston, UK.

## Abstract

**Images:**


Br. J. Cancer (1988), 57, 559-563 © The Macmillan Press Ltd., 1988

### Near neighbour analysis of variant cell lines derived from the promyeloid cell line HL60

C.M. Bunce, J.M. Lord, A.K.-Y. Wong & G. Brown

*Department of Immunology, University of Birmingham, Edgbaston, Birmingham B15 2TJ, UK.*

**Summary** The human promyeloid cell line H60 can be induced to differentiate towards either neutrophils or monocytes. Variant cell lines, derived from HL60, which show reduced capacities for neutrophil and monocyte differentiation can be arranged in a developmental sequence which suggests that the potentials for neutrophil and monocyte differentiation are expressed sequentially by HL60 cells in this order. Analysis of the patterns of total cellular phosphoproteins within HL60 and 5 variant cell lines, by two-dimensional gel electrophoresis, has identified 6 distinct phosphoproteins which show progressive differences in the intensity of spots between the variant lines. The changes in these phosphoproteins relate to the position of the lines within the proposed development sequence. Similarly, lines placed close together in the sequence are more similar, as regards phosphoprotein profiles, than lines placed far apart. These studies provide direct evidence in favour of the hypothesis that the potentials for neutrophil and monocyte differentiation are expressed sequentially during myelopoiesis. Furthermore, two phosphoprotein spots were found to be restricted to lines able to differentiate towards monocytes. These proteins may play important roles during commitment to monocyte differentiation.

A central problem of cell biology is to discover how any multipotent stem cell, from the fertilised egg onwards, becomes progressively more committed towards a particular pathway of differentiation. A valuable model system for studies of cell commitment is provided by the haemopoietic system in that pluripotent stem cells give rise to at least five distinct cell types. As yet, it is not clear whether haemopoietic stem cells can be directly committed to differentiate along each maturation pathway or whether there is a preferred course of stem cell development in which lineage options are expressed in a particular order. For example, Greaves and co-workers have proposed that early lineage-associated markers are co-expressed (Greaves *et al.*, 1986) which suggests that five lineage options may co-exist. Alternatively, it can be argued that progenitor cells develop lineage potentials sequentially (Brown *et al.*, 1985; 1987) and, for example, during myelopoiesis cells first acquire the capacity for neutrophil maturation and are subsequently able to differentiate towards monocytes (Brown *et al.*, 1985; Dexter *et al.*, 1980).

We have used the human promyeloid cell line HL60, which can be induced to mature towards either neutrophils (Collins *et al.*, 1978) or monocytes (Rovera *et al.*, 1979), to investigate cell commitment. Our approach in using HL60 cells to study the cellular processes which control commitment has been to derive variant lines which show reduced capacities for neutrophil and monocyte differentiation (Toksoz *et al.*, 1982; Bunce *et al.*, 1983). The lines were then studied in detail as regards their relative responsiveness to inducers of neutrophil and monocyte differentiation and their expression of myeloid cell surface antigens (Bunce *et al.*, 1983). By using these data, the variant lines can be arranged in a linear developmental sequence as regards expression of the potentials for neutrophil and monocyte differentiation (Brown *et al.*, 1985). In this model, HL60 cells first acquire the capacity for granulocyte differentiation, subsequently they develop the ability to respond to inducers of monocyte differentiation and at a later stage are restricted to monocyte differentiation (Brown *et al.*, 1985). Thus, the variant HL60 cell lines typify cells within HL60 cultures at slightly different stages of development with respect to commitment to neutrophil and monocyte differentiation. If the above notion, and the developmental ordering of the variant HL60 cell lines are correct, then near-neighbour comparisons of the proteins of each of the lines, by two-dimensional gel electrophoresis, should verify the hypothesis. In other words, the protein patterns of lines which are near-neighbours in the proposed developmental sequence should display the greatest similarity and the patterns obtained for the lines placed far apart in the sequence be least similar. Progressive differences in the protein patterns of each of the lines showing that the lines have been placed in a correct order of developmental would provide direct evidence in favour of a sequential model of commitment to neutrophil and monocyte differentiation.

If the variant lines typify cells at slightly different stages of commitment, then the panel of lines provides a series of stable 'windows' through which we can view early events during cell commitment. Thus, comparative analyses of the variant lines should identify those proteins which regulate key genes during commitment to neutrophil and monocyte differentiation. The discovery that oncogene-encoded proteins and several growth factor receptors display tyrosine kinase activity has suggested that protein phosphorylation is important in regulating the function of many proteins that are involved in cell growth and differentiation (Bishop, 1983). Therefore, in analyses of the variant cell lines, attention was focussed on phosphoproteins. In the present work, the parental HL60 cells and five variant lines have been subjected to near-neighbour analysis by examining the patterns obtained, by two-dimensional gel electrophoresis, for total cellular phosphoproteins.

### Materials and methods

### Cell lines

The promyeloid cell line HL60 was maintained in RPMI 1640 medium (Gibco Ltd., Paisley, Scotland) supplemented with 10% v/v heat inactivated foetal calf serum (Gibco Ltd., Paisley, Scotland), 100 U $ml^{-1}$ penicillin and 100 $\mu g\, ml^{-1}$ streptomycin (Gibco Ltd., Paisley, Scotland). The isolation and characterisation of the variant HL60 cell lines HL60M2, HL60M4, HL60 Ast3 and HL60 Ast4 have been described previously (Toksoz *et al.*, 1982; Bunce *et al.*, 1983). The variant line HL60 15-12 was a gift from Dr A.G. Fisher (NIH, Bethesda). All the variant lines were maintained in the above medium containing 1.25% dimethylsulphoxide (DMSO). This concentration of DMSO induces optimal neutrophil differentiation within HL60 cultures. Neutrophil differentiation is induced in cultures of HL60M2, HL60M4, HL60 Ast3 and HL60 15-12 cells by 1.75% DMSO and HL60 Ast4 cultures show minimal neutrophil

Correspondence: G. Brown.
Received 17 October 1987; and in revised form, 5 January 1988.

differentiation when treated with 2.0% DMSO. Cells within HL60 Ast3 and HL60 Ast4 cultures are unable to differentiate towards monocytes when treated with 12-0-tetradecanoylphorbol-13-acetate (TPA) (Bunce et al., 1983).

*Labelling of cells with [$^{32}P$]-orthophosphate*

HL60 or variant HL60 cells ($1.5 \times 10^6$) were pelleted (150 g, 10 min at room temperature) and resuspended in 300 $\mu$l RPMI 1640 medium supplemented with 2 mg ml$^{-1}$ bovine serum albumin (Fraction V, Sigma, Poole, UK) which had been dialysed against ethylenediaminetetraacetic acid (EDTA) and then neutralised to pH 7. Basic media such as Lockes, Minimal essential medium and phosphate-free media were not used since HL60 cells incubated in these media showed poor cell viability as revealed by phase contrast microscopy and a rapid decline in cellular ATP levels over a 6 h period. Cells were pre-incubated for 15 min at 37°C when [$^{32}$P]-orthophosphate at 10 mCi ml$^{-1}$, pH 7 (Amersham International plc, Amersham, UK) was added to give a final activity of 0.5 mCi ml$^{-1}$. The cells were then incubated at 37°C for a further 5 h. This labelling time was required to allow extracellular [$^{32}$P]-orthophosphate to reach equilibrium with intracellular [$^{32}$P]-ATP. The incubation was terminated by adding 200 $\mu$l cell suspension to 1 ml of ice cold RPMI 1640 medium and the cells pelleted rapidly by centrifugation for 1 min in a microfuge (Beckman model B, Beckman-RIIC Ltd., High Wycombe, UK). The cell pellet was resuspended in 80 $\mu$l electrophoresis sample buffer containing 9 M urea, 2% 3-[(3-cholamidopropyl) dimethylammonio] 1-propane sulphonate (CHAPS), 5% $\beta$-mercaptoethanol, 2% ampholines pH 3.5–10 (LKB, Croydon, UK), 100 $\mu$g ml$^{-1}$ butylated hydroxytoluene and 4% NP40.

*Two-dimensional gel electrophoresis*

A 75 $\mu$l aliquot of each sample was analysed by two-dimensional gel electrophoresis, essentially as described by O'Farrell (1975) but using a multiple isoelectric focussing apparatus in the first dimension (Anderson & Anderson, 1978). Briefly, isoelectric focussing (IEF) was performed for 15 h at 700 volts in 130 × 1.5 mm 4% polyacrylamide gels containing 2% ampholines [1.6% pH 3–10 (LKB); 0.4% pH 4–6 (LKB)]. The second dimension was SDS polyacrylamide gel electrophoresis (10% separating gel; 5% stacking gel). Molecular weights and pI values were determined by comparison with known standard proteins (Sigma, Poole, UK). Gels were silver stained (Oakley et al., 1980) and dried prior to autoradiography. Films (Hyper-MP X-ray film, Amersham International plc, Amersham, UK) were exposed for 3–4 days at −70°C with intensifying screens. Autoradiographs were analysed by scanning densitometry using an LKB ultroscan. In order to ensure reproducibility of the autoradiographs, HL60 cells and the five variant cell lines were [$^{32}$P] labelled, extracted and electrophoresed in parallel. The data shown is representative of five separate experiments in which consistent results were obtained.

### Results

The phosphoproteins patterns observed in autoradiographs of two-dimensional gels prepared from [$^{32}$P]-orthophosphate labelled HL60 and variant HL60 cells were complex. As shown in Figure 1, more than 150 distinct phosphoproteins can be readily seen in the patterns obtained for HL60 and HL60 Ast3 cells. Despite this complexity, the phosphoprotein patterns for HL60 and each of the variant lines were reproducible in five separate experiments. Furthermore, the patterns of phosphoproteins obtained for the five variant lines were almost identical to the pattern obtained for HL60 cells (as shown for HL60 Ast3 in Figure 1). Careful visual and densitometric analyses of the autoradiographs revealed only six distinct phosphoproteins which showed clear differences in their level of expression or degree of phosphorylation within HL60 cells and the variant cell lines. Differences in the six proteins were consistently observed in all five experiments.

Selected areas of autoradiographs which focus attention on the differences between HL60 and the variant cell lines HL60 Ast4, HL60 Ast3 and HL60 15-12 are shown in Figure 2. With respect to the phosphoproteins of interest, the patterns obtained for HL60M2 and HL60M4 (data not shown) were similar to that of HL60 15-12. The order of the variant lines as regards their acquisition of an increased capacity for neutrophil differentiation, followed by loss of this capacity as cells are committed to monocyte differentiation, is as follows: HL60 Ast4, HL60 Ast3, HL60 and HL60M2, HL60M4, HL60 15-12 are at the same stage of development. Two phosphoproteins with molecular weight and pI values of 48 kD, 5.0 and 29 kD, 6.0 (see a and c, Figure 2) were observed as intense spots in autoradiographs

**Figure 1** Two dimensional gel electrophoresis (IEF) of [$^{32}$P] orthophosphate-labelled proteins from HL60 and HL60 Ast3 cells. The area enclosed by the broken lines indicates the areas of the autoradiographs considered in detail in **Figure 2**. The spots labelled $S_1$ and $S_2$ are reference spots used in the densitometric analysis.

Figure 2 Comparative analysis of selectedc areas of two dimensional gels of [$^{32}$P]-orthophosphate-labelled proteins from HL60 and the variant cells HL60 Ast4, HL60 Ast3 and HL60 15-12. There are 6 distinct proteins which show clear differences in the intensity of the spots. These have molecular weight and pI values as follows: a, 48 kD/5.0; b, 29 kD/6.6; c, 29 kD/6.0; d, 15 kD/6.6; e, 15 kD/3.3; and f, 13 kD/4.6.

prepared using HL60 Ast4 cell extracts. Both spots showed a reduced intensity in the case of HL60 Ast3 cells and were greatly reduced in intensity in autoradiographs of HL60, HL60M2, HL60M4 and HL60 15-12 cell extracts. The 48 kD, 5.0 phosphoprotein was always observed as a streak on autoradiographs prepared from HL60 and the variant cell extracts. Therefore, the molecular weight and isoelectric point values given for this phosphoprotein are approximate. Indeed, the streaked appearance of the spot may be due to the presence of more than one phosphoprotein which have not been adequately resolved in the gel system used. Two phosphoproteins having molecular weight and pI values of 29 kD, 6.6 (see b, Figure 2) and 15 kD, 6.6 (see d, Figure 2) were present as clear spots in autoradiographs of HL60 Ast4 and HL60 Ast3 cell extracts and the intensity of the spots was reduced in the case of HL60 cells and the remaining HL60 sub-lines.

The four phosphoproteins described above were readily detected in the two variant cell lines, HL60 Ast4 and HL60 Ast3, which are postulated to represent early stages in the developmental sequence. In contrast to these phosphoproteins, two phosphoproteins with molecular weight and pI values of 15 kD, 3.3 (see e, Figure 2) and 13 kD, 4.6 (see f, Figure 2) were readily detected in autoradiographs of HL60, HL60M2, HL60M4 and HL60 15-12 cells but were absent or just visible in autoradiographs of HL60 Ast4 and HL60 Ast3 cell extracts (see Figure 2). The HL60, HL60M2, HL60M4 and HL60 15-12 lines are able to differentiate towards monocytes and the HL60 Ast4 and HL60 Ast3 cells are postulated to represent a developmental stage prior to acquisition of this potential.

The autoradiographs obtained for each of the lines were analysed by scanning densitometry. In order to eliminate the possibility that the differences seen in the patterns of phosphoproteins were due to unequal sample loading of the IEF gels, phosphoproteins which gave readily identifiable and reproducible spots on autoradiographs from all the cell lines were used as reference standards (see S1, S2, Figure 1). In comparative analyses of the HL60 and the variant lines the densitometric values obtained for the six phosphoproteins of interest were normalised with respect to the absorbance readings for the standard spots. The results, which are qualitative at this stage, are summarised in Figure 3 and confirm the differences in the phosphoprotein pattern observed between the HL60 and variant cell lines.

| Phosphoprotein Mr | pI | HL60 Ast4 | HL60 Ast3 | HL60 | HL60 M2, 15–12, M4 |
|---|---|---|---|---|---|
| a 48 kd | 5.0 | +++ | ++ | + | + |
| b 29 kd | 6.6 | +++ | +++ | + | + |
| c 29 kd | 6.0 | +++ | ++ | +/− | +/− |
| d 15 kd | 6.6 | ++ | ++ | + | + |
| e 15 kd | 3.3 | − | − | + | + |
| f 13 kd | 4.6 | − | − | + | + |

Figure 3 Relationship between the differences in phosphoproteins observed and the positions of the variant cell lines in the postulated developmental sequence. The lines are arranged in sequence as regards acquisition of the potential for neutrophil differentiation [ ◎ ) and their increased sensitivity to inducers of neutrophil differentiation ( ↗ ) followed by loss of this responsiveness ( ↘ ) as cells acquire the potential for monocyte differentiation (Ⓜ). The relative intensities of the spots were scored in relation to densitometric readings.

To investigate whether the differences in phosphoprotein spots seen between the lines related to differences in the level of protein expression or their degree of phosphorylation, autoradiographs obtained for each of the lines were compared with the silver stained gels. However, no silver stained spots coincided with the positions of the 29 kD, 15 kD or 13 kD phosphoproteins (see Figure 4). Therefore these phosphoproteins are present at low levels within HL60 cells. A minor protein was detected in the silver stained gels which was coincident with the position of the 48 kD phosphoprotein streak. However, this slver stained protein which lies in the left hand portion of the streak does not show a streaked appearance and is unlikely to wholly represent the 48 kD phosphoprotein. Since the phosphoproteins of interest cannot be readily identified on the silver stained two-dimensional gels, it has not been possible to determine whether differences in phosphoprotein patterns between the HL60 sublines is due to variations in protein levels or the degree to which each of the proteins is phosphorylated. Resolution of this problem will require extensive two-dimensional gel analysis of subcellular fractions which may reveal the silver stained proteins which correlate with the phosphoprotein spots.

### Discussion

The aim in this study was to investigate whether a panel of HL60 variant cell lines had been arranged in a correct developmental order which postulated that the potentials for neutrophil and monocyte differentiation are expressed sequentially. The variant cell lines had been ordered in relation to their gradual acquisition of an increased responsiveness to inducers of neutrophil differentiation followed by loss of this potential as cells are committed to monocyte differentiation [Brown *et al.*, 1985 and see Figure 3]. The predictions of this model were twofold. First, that the protein patterns obtained for lines placed close together in the sequence (HL60 Ast4 and HL60 Ast3) should be more similar than the patterns for lines placed far apart (HL60 Ast4 and HL60 15-12). Secondly, that there should be

**Figure 4** Two dimensional gel electrophoresis (IEF) of total proteins from HL60 and HL60 Ast4 cells as revealed by silver staining. The positions of the six phosphoproteins, which show differences between the various cell lines, were marked by overlaying the silver stained gels and autoradiographs.

gradual, progressive changes in the protein profiles for each of the lines in keeping with their position in the sequence.

Analysis of the patterns obtained for whole cell phosphoproteins of HL60 cells and the variant cell lines has revealed a differential pattern of intensity of six phosphoprotein spots which fulfils both the above predictions. With respect to the six phosphoproteins, the pattern of HL60 Ast4 cells is most similar to that of HL60 Ast3 and least closely related to that of HL60 15-12 (see Figure 3). No differences were observed as to the presence or absence of the six phosphoproteins in the case of HL60 Ast4 and HL60 Ast3 cells, the intensities of all six phosphoprotein spots were clearly different in the case of HL60 Ast4 and HL60 15-12. Furthermore, the patterns of the six phosphoproteins are near identical in the case of HL60M2, HL60M4 and HL60 15-12 which are placed at the same stage in the developmental sequence. These three lines are independent sublines of HL60 in that the HL60 15-12 line was derived in Washington and the HL60M2 and HL60M4 lines were derived in our own laboratory from separate stocks of the parental HL60 cells. Karyotype analyses of the HL60M2 and HL60M4 lines have shown that these lines have distinctive karyotypes (A.M.R. Taylor, unpublished observation).

Gradual and progressive changes in the levels of intensity of phosphoprotein spots were observed in the case of two phosphoproteins. Both the 48 kD, pI 5.0 and 29 kD, pI 6.0 phosphoprotein spots showed a progressive diminution throughout the lines (see Figure 3). Densitometric readings obtained for the 48 kD, pI 5.0 protein for the lines HL60 Ast4, HL60 Ast3, HL60 and HL60 15-12 were 0.64, 0.38, 0.19 and 0.13 respectively. Values obtained for these lines in the case of the 29 kD, pI 6.0 protein were 1.02, 0.52, 0.27 and 0.05. These gradual changes observed within the lines argue in favour of placing the lines in a linear sequence, in the proposed order. The linear sequence favours the hypothesis that the potentials for neutrophil and monocyte differentiation are expressed sequentially during myelopoiesis (Brown et al., 1985, 1987) which is in agreement with a previous suggestion by Dexter and colleagues that the sequence of development of normal myeloid colony forming cells (CFC) is G-CFC→GM-CFC→M-CFC (Dexter et al., 1980).

The differences observed between the HL60 sub-lines were few and correspond to minor proteins within cells. It is predictable that within this group of proteins are key proteins which regulate commitment to neutrophil or monocyte differentiation (see **Introduction**). Of particular interest are the 15 kD, pI 3.3 and 13 kD, pI 4.6 phosphoprotein spots that appear in cells which are able to respond to inducers of monocyte differentiation. These proteins may play important roles in commitment along this pathway of maturation. At present, it is only possible to speculate about the possible roles of the phosphoproteins identified in this study. The varied molecular weights and pI values of the phosphoprotein suggest that they perform a variety of functions. The observed differences in phosphoprotein patterns in relation to the developmental status of the variant cells suggests that the availability of substrates for protein kinases could influence the lineage potential of cells. However, this also poses the question of availability of appropriate kinases. Protein kinase C has been implicated in the differentiation of HL60 cells towards monocytes (Ebeling et al., 1985; Vandenbark et al., 1984) and two groups have recently described multiple protein kinase C genes (Knopf et al., 1986; Coussens et al., 1986). It is interesting to speculate that the sequential expression or activation of particular protein kinases C and/or other protein kinases and their co-ordinated action may play a vital role in the commitment of HL60 cells to one pathway or another of differentation.

If the phosphoproteins identified by comparative analysis of the variant cell lines do play important roles during HL60 commitment to neutrophil and monocyte differentiation then they should also change their phosphorylation status when HL60 cells are induced to mature along the above pathways. Detailed dose response and time course experiments have been undertaken to investigate this possibility when HL60 cells were induced to mature towards monocytes by TPA (Lord et al., 1988). This agent is known to be an activator of protein kinase C (Castraga et al., 1982). One of the six phosphoproteins, the 15 kD, pI 6.6 phosphoprotein spot, showed an increased level of phosphorylation when HL60 cells were treated for 10 minutes with an amount of TPA (10 nM) optimal for the induction of differentiation (Lord et al., 1988). Interestingly, this reveals a paradox which is that the 15 kD protein is constitutively phosphorylated in variant cell lines which are unable to differentiate towards monocytes and that phosphorylation of this protein may play a role in monocyte differentiation. One explanation of these data is that the protein is phosphorylated in the variant lines unable to mature towards monocytes at one site which inhibits the activity of the protein which is required during commitment to monocyte differentiation. Subsequent dephosphorylation and phosphorylation at a second site confers functional activity on the protein which plays a role during monocyte differentiation. Further, detailed studies will be required to investigate whether any of the five remaining proteins change their phosphorylation status when HL60 cells are treated with inducers of neutrophil differentiation. Other than substrates for protein kinases, one or more of the six phosphoproteins identified in this study may be either protein kinases themselves or receptors whose activity is modulated by autophosphorylation (Sibley *et al.*, 1987).

The impoprtant question addressed in this study was whether, within HL60 cultures, there is a sequential development of cells expressing potentials for neutrophil and monocyte differentiation or whether cells can be committed directly along either pathway of maturation. Three separate areas of investigation now accord and argue in favour of the first proposal. The relative responsiveness of the variant cell lines to inducers of neutrophil and monocyte differentiation suggested that cells within HL60 show a gradual aquisition of the ability to respond to inducers of neutrophil differentiation followed by loss of this potential as cells acquire the potential for monocyte differentiation (Brown *et al.*, 1985). This observation led to the suggestion that the lines typify a linear sequence of commitment (Brown *et al.*, 1985). Variant cell lines have been described which show variable capacities for neutrophil differentiation and which are either able or unable to mature towards monocytes (Toksoz *et al.*, 1982; Bunce *et al.*, 1983). This pattern of responsiveness cannot readily be explained by the model whereby cells can be directly committed along either pathway of differentiation. The position of the lines in a linear sequence is then confirmed by two further observations. As argued previously, variable expression of two myeloid-associated antigens by variant lines, in relation to the expression of these antigens by mature neutrophils and monocytes, can be explained by the position of lines in the sequence (Brown *et al.*, 1985). Furthermore, consideration of myeloid antigen expression has suggested that the potential for neutrophil differentiation is expressed prior to the potential for monocyte differentiation (Brown *et al.*, 1985). In this study, we have confirmed the close relationships of variant lines, which is suggested by a linear sequence of commitment, by demonstrating that the number of differences observed in phosphoproteins and progressive changes relate to whether the lines are placed close together or far apart in the linear sequence.

In conclusion, the above considerations, taken together, argue that lineage potentials are expressed sequentially and in a predetermined manner during myelopoiesis (Brown *et al.*, 1985; Brown *et al.*, 1987). This process may not be restricted to myeloid progenitor cell development but may be a general mechanism of haemopoietic cell development (Brown *et al.*, 1985; Brown *et al.*, 1987). The use of near-neighbour analysis of variant lines derived from HL60 has also enabled us to identify six phosphoproteins which may play vital roles during myelopoiesis. The precise nature of these proteins and their distribution, in particular, whether any of the proteins are located within the nucleus and directly regulate key genes, are of particular interest. The analysis illustrates the usefulness of variant cell lines in studies of the mechanisms of lineage determination.

We thank the Leukaemia Research Fund and The Medical Research Council, UK for support of research in our laboratory. Amanda K.-Y. Wong is a recipient of a scholarship and research training support grant from The Croucher Foundation, Kowloon, Hong Kong. We are grateful to Petra Hickey for typing the manuscript.

### References

ANDERSON, N.G. & ANDERSON, N.L. (1978). Analytical techniques for cell fractions. XXI. Two-dimensional analysis of serum and tissue proteins: Multiple isoelectric focussing. *Anal. Biochem.*, **85**, 331.

BISHOP, J.M. (1983). Cellular oncogenes and retroviruses. *Ann. Rev. Biochem.*, **52**, 301.

BROWN, G., BUNCE, C.M. & GUY, G.R. (1985). Sequential determination of lineage potentials during haemopoiesis. *Br. J. Cancer*, **52**, 681.

BROWN, G., BUNCE, C.M., HOWIE, A.J. & LORD, J.M. (1987). Stochastic or ordered lineage commitment during haemopoiesis. *Leukaemia*, **1**, 150.

BUNCE, C.M., FISHER, A.G., TOKSOZ, D. & BROWN, G. (1983). Isolation and characterisation of dimethylsulphoxide resistant variants from the human promyeloid cell line HL60. *Exp. Hematol.*, **11**, 828.

CASTAGRA, M., TAKAI, Y., KAIBUCHI, K., SANO, K., KIKKAWA, U. & NISHIZUKA, Y. (1982). Direct activation of calcium-activated, phospholipid-dependent protein kinase by tumour-promoting phorbol esters. *J. Biol. Chem.*, **257**, 7847.

COLLINS, S.J., RUSCETTI, F.W., GALLAGHER, R.E. & GALLO, R.C. (1978). Terminal differentiation of human promyelocytic cells induced by dimethyl sulphoxide and other polar compounds. *Proc. Natl Acad. Sci. USA.*, **75**, 2458.

COUSSENS, L., PARKER, P.J., RHEE, L. & 5 others (1986). Multiple, distinct forms of bovine and human protein kinase C suggest diversity in cellular signalling pathways. *Science*, **233**, 859.

DEXTER, T.M., GARLAND, J., SCOTT, D., SCOLNICK, E. & METCALF, D. (1980). Growth of factor-dependent hemopoietic precursor cell lines. *J. Exp. Med.*, **152**, 1036.

EBELING, J.G., VANDENBARK, G.R., KUHN, L.J., GANONG, B.R., BELL, R.M. & NIEDEL, J.E. (1985). Diacyglycerols mimic phorbol diester induction of leukaemia cell differentiation. *Proc. Natl Acad. Sci. USA.*, **73**, 448.

GREAVES, M.F., CHAN, L.C., FURLEY, A.J.W., WATT, S.M. & MOLGAARD, H.V. (1986). Lineage promiscuity in haemopoietic differentiation and leukaemia. *Blood*, **67**, 1.

KNOPF, J.L., LEE, M.H., SULTZMAN, L.A. & 4 others (1986). Cloning and expression of multiple protein kinase C cDNA's. *Cell.*, **46**, 491.

LORD, J.M., WONG, A.K.-Y. & BROWN, G. (1988). Changes in phosphoproteins during commitment of HL60 cells to monocyte differentiation: Evidence for multiple protein kinase involvement. *Exp. Hematol.* (In press).

OAKLEY, B.R., KIRSCH, D.R. & MORRIS, N.R. (1980). A simplified ultrasensitive silver stain for detecting proteins in polyacrylamide gels. *Anal. Biochem.*, **105**, 361.

O'FARRELL, P.H. (1975). High resolution two-dimensional electrophoresis of proteins. *J. Biol. Chem.*, **250**, 4007.

ROVERA, G., O'BRIEN, T.G. & DIAMOND, L. (1979). Induction of differentiation in human promyelocytic leukaemia cells by tumour promotors. *Science*, **204**, 868.

SIBLEY, O.R., BENOVIC, J.L., CARAN, M.G. & LEFKOWITZ, R.J. (1987). Regulation of transmembrane signalling by receptor phosphorylation. *Cell*, **48**, 913.

TOKSOZ, D., BUNCE, C.M., STONE, P.C.W., MICHELL, R.H. & BROWN, G. (1982). Variant cell lines from the human promyelocyte line HL60. *Leukemia Res.*, **6**, 491.

VANDENBARK, G.R., KUHN, L.J. & NIEDEL, J.E. (1984). Possible mechanism of phorbol ester-induced maturation of human promyelocytic leukaemia cells. *J. Clin. Invest.*, **73**, 448.


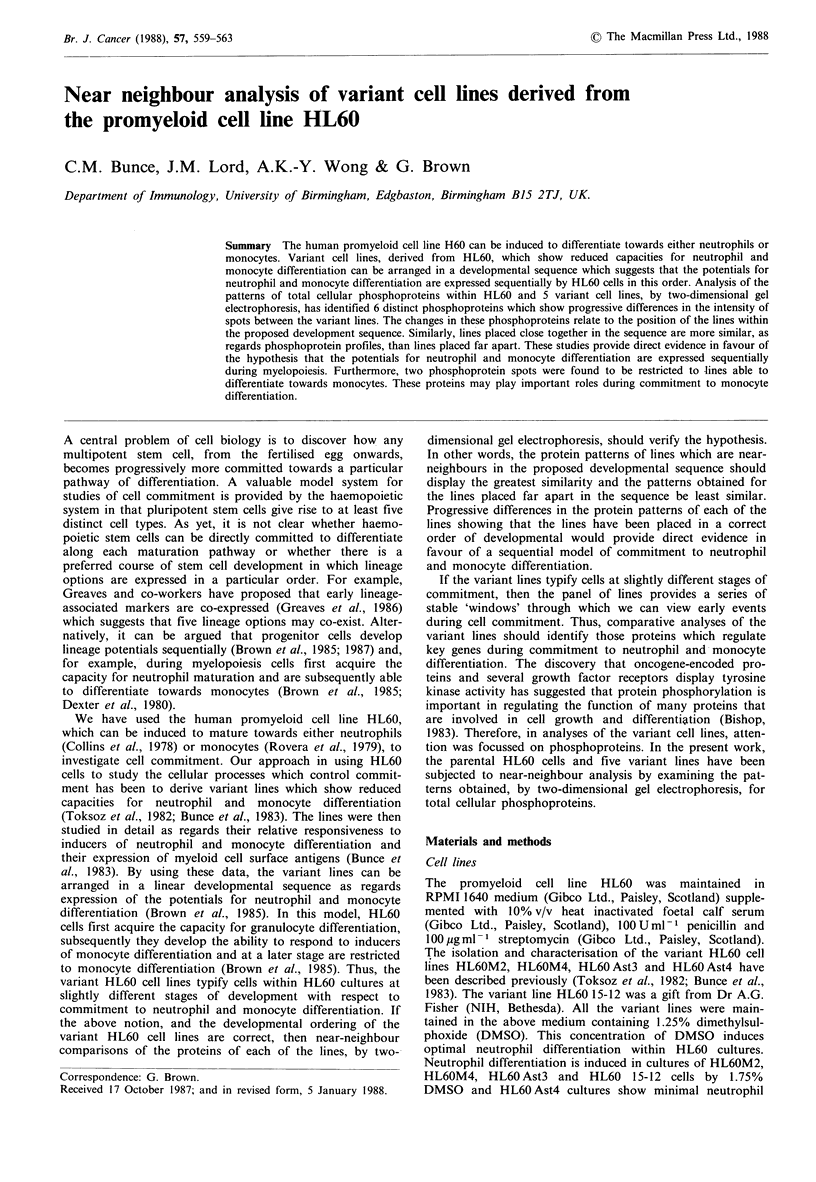

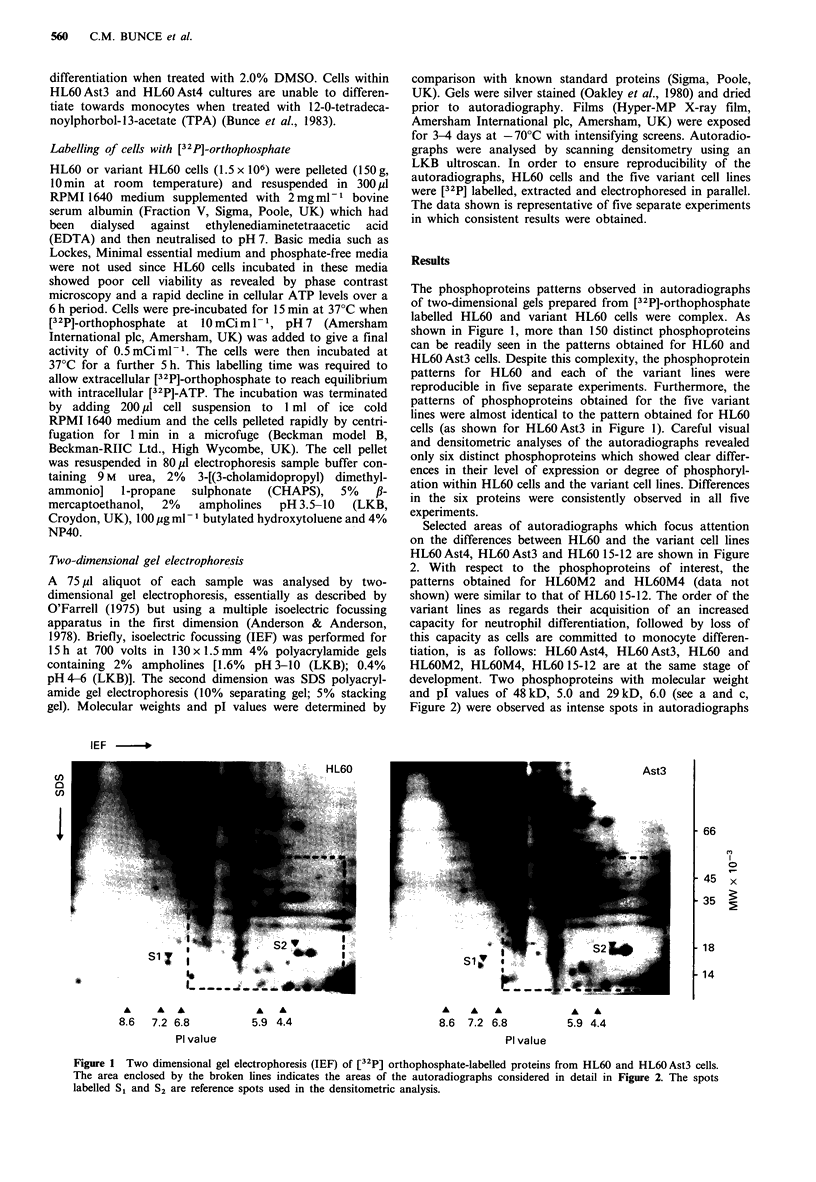

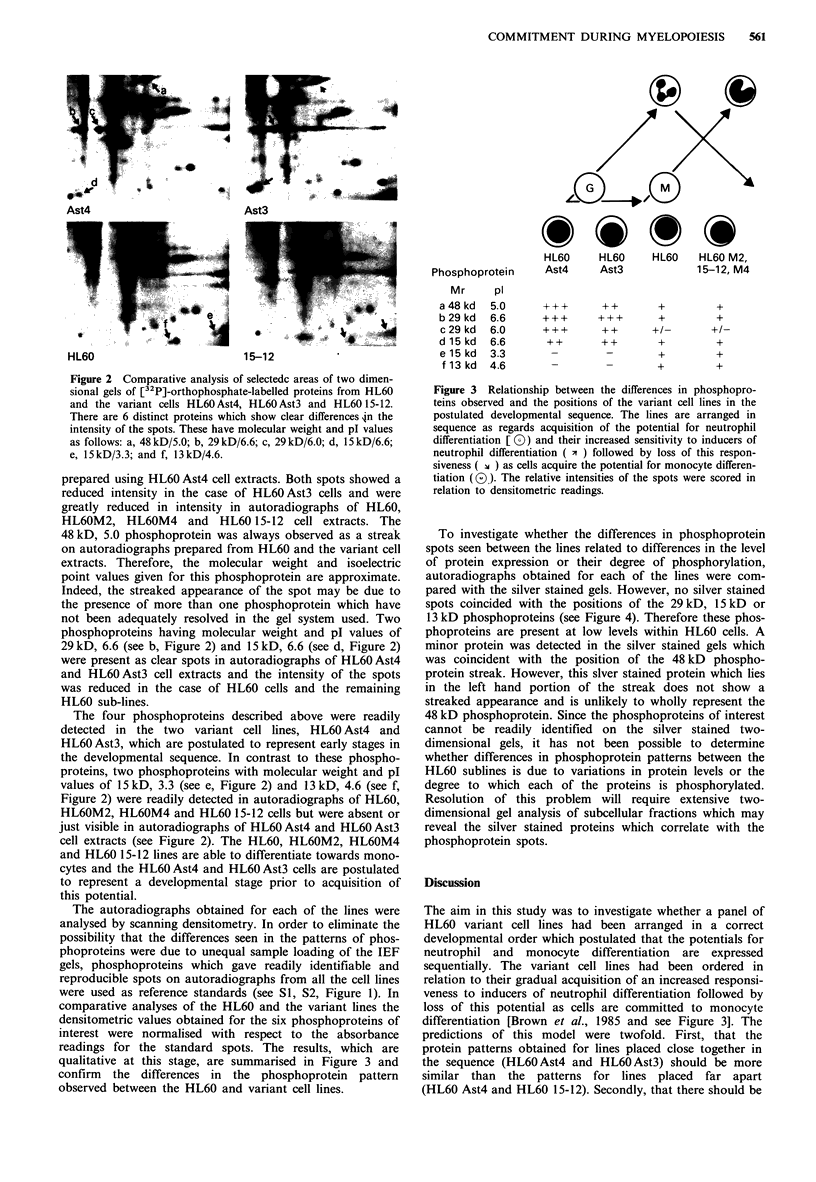

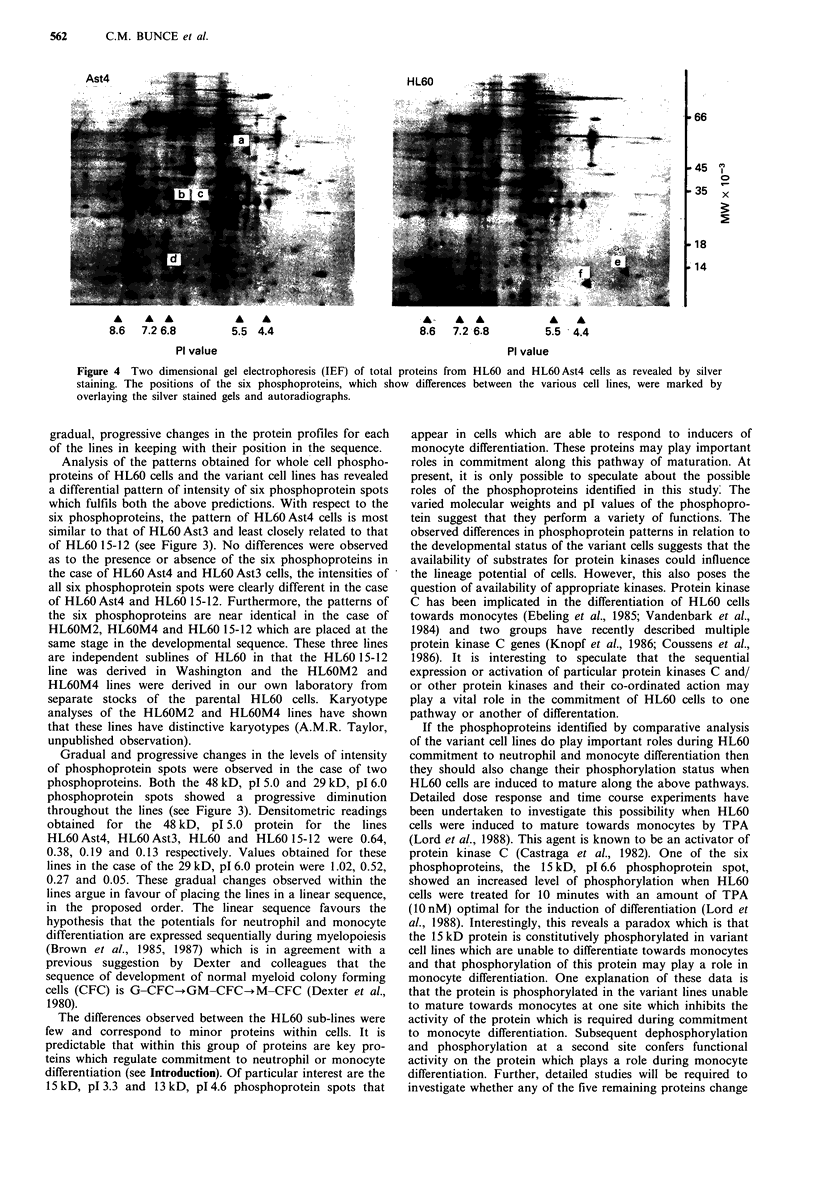

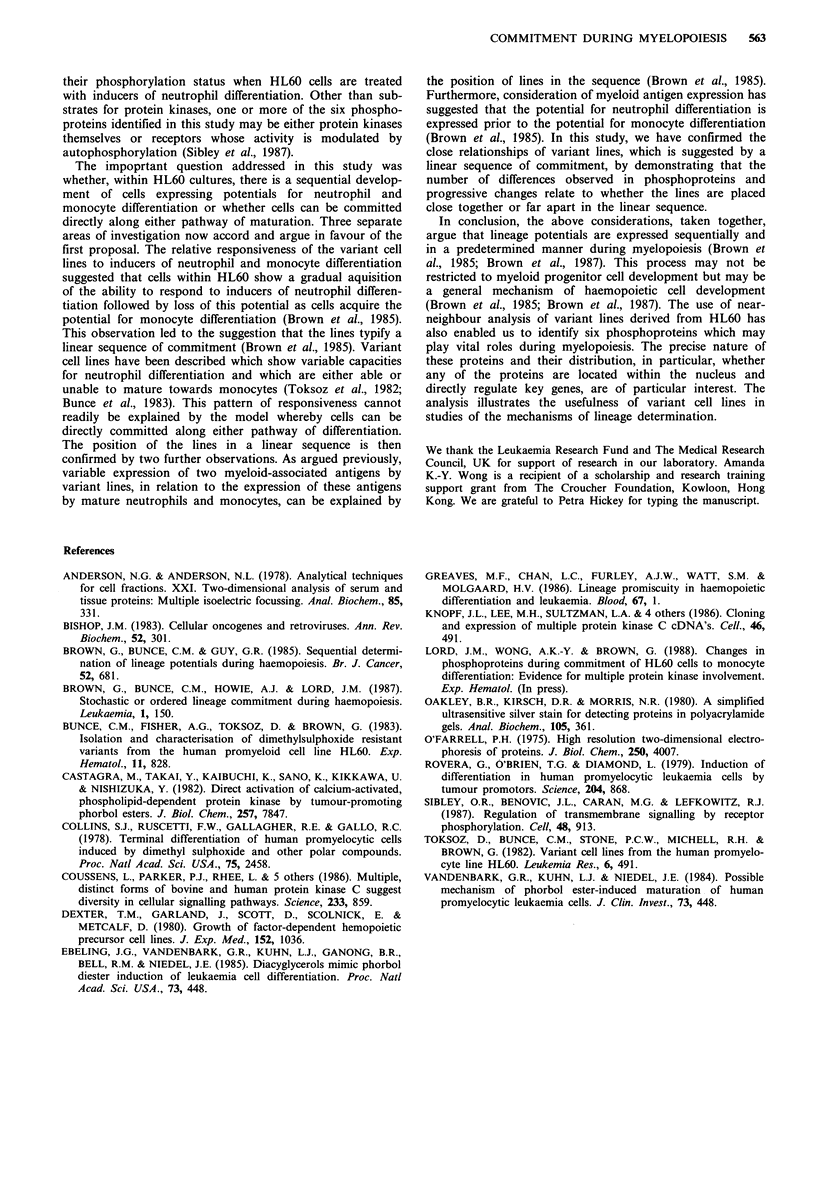


## References

[OCR_00668] Anderson N. G., Anderson N. L. (1978). Analytical techniques for cell fractions. XXI. Two-dimensional analysis of serum and tissue proteins: multiple isoelectric focusing.. Anal Biochem.

[OCR_00674] Bishop J. M. (1983). Cellular oncogenes and retroviruses.. Annu Rev Biochem.

[OCR_00678] Brown G., Bunce C. M., Guy G. R. (1985). Sequential determination of lineage potentials during haemopoiesis.. Br J Cancer.

[OCR_00683] Brown G., Bunce C. M., Howie A. J., Lord J. M. (1987). Stochastic or ordered lineage commitment during hemopoiesis?. Leukemia.

[OCR_00688] Bunce C. M., Fisher A. G., Toksoz D., Brown G. (1983). Isolation and characterisation of dimethylsulphoxide resistant variants from the human promyeloid cell line HL60.. Exp Hematol.

[OCR_00694] Castagna M., Takai Y., Kaibuchi K., Sano K., Kikkawa U., Nishizuka Y. (1982). Direct activation of calcium-activated, phospholipid-dependent protein kinase by tumor-promoting phorbol esters.. J Biol Chem.

[OCR_00700] Collins S. J., Ruscetti F. W., Gallagher R. E., Gallo R. C. (1978). Terminal differentiation of human promyelocytic leukemia cells induced by dimethyl sulfoxide and other polar compounds.. Proc Natl Acad Sci U S A.

[OCR_00706] Coussens L., Parker P. J., Rhee L., Yang-Feng T. L., Chen E., Waterfield M. D., Francke U., Ullrich A. (1986). Multiple, distinct forms of bovine and human protein kinase C suggest diversity in cellular signaling pathways.. Science.

[OCR_00711] Dexter T. M., Garland J., Scott D., Scolnick E., Metcalf D. (1980). Growth of factor-dependent hemopoietic precursor cell lines.. J Exp Med.

[OCR_00722] Greaves M. F., Chan L. C., Furley A. J., Watt S. M., Molgaard H. V. (1986). Lineage promiscuity in hemopoietic differentiation and leukemia.. Blood.

[OCR_00727] Knopf J. L., Lee M. H., Sultzman L. A., Kriz R. W., Loomis C. R., Hewick R. M., Bell R. M. (1986). Cloning and expression of multiple protein kinase C cDNAs.. Cell.

[OCR_00743] O'Farrell P. H. (1975). High resolution two-dimensional electrophoresis of proteins.. J Biol Chem.

[OCR_00738] Oakley B. R., Kirsch D. R., Morris N. R. (1980). A simplified ultrasensitive silver stain for detecting proteins in polyacrylamide gels.. Anal Biochem.

[OCR_00747] Rovera G., O'Brien T. G., Diamond L. (1979). Induction of differentiation in human promyelocytic leukemia cells by tumor promoters.. Science.

[OCR_00752] Sibley D. R., Benovic J. L., Caron M. G., Lefkowitz R. J. (1987). Regulation of transmembrane signaling by receptor phosphorylation.. Cell.

[OCR_00757] Toksoz D., Bunce C. M., Stone P. C., Michell R. H., Brown G. (1982). Variant cell lines from the human promyelocyte line HL60.. Leuk Res.

[OCR_00762] Vandenbark G. R., Kuhn L. J., Niedel J. E. (1984). Possible mechanism of phorbol diester-induced maturation of human promyelocytic leukemia cells.. J Clin Invest.

